# Evaluation of Out-of-Pocket Costs and Treatment Intensification With an SGLT2 Inhibitor or GLP-1 RA in Patients With Type 2 Diabetes and Cardiovascular Disease

**DOI:** 10.1001/jamanetworkopen.2023.17886

**Published:** 2023-06-12

**Authors:** Jing Luo, Robert Feldman, Katherine Callaway Kim, Scott Rothenberger, Mary Korytkowski, Inmaculada Hernandez, Walid F. Gellad

**Affiliations:** 1 Division of General Internal Medicine, Department of Medicine, University of Pittsburgh School of Medicine, Pittsburgh, Pennsylvania; 2 Graduate School of Public Health, University of Pittsburgh, Pittsburgh, Pennsylvania; 3 Division of Endocrinology and Metabolism, University of Pittsburgh School of Medicine, Pittsburgh, Pennsylvania; 4 Skaggs School of Pharmacy and Pharmaceutical Sciences, University of California, San Diego, La Jolla; 5 Center for Health Equity Research and Promotion, VA Pittsburgh Healthcare System, Pittsburgh, Pennsylvania

## Abstract

**Importance:**

The latest guidelines continue to recommend sodium-glucose cotransporter 2 (SGLT2) inhibitors and glucagon-like peptide-1 receptor agonists (GLP-1 RAs) for patients with type 2 diabetes (T2D) and established cardiovascular disease (CVD). Despite this, overall use of these 2 drug classes has been suboptimal.

**Objective:**

To assess the association of high out-of-pocket (OOP) costs and the initiation of an SGLT2 inhibitor or GLP-1 RA among adults with T2D and established CVD who are treated with metformin-treated.

**Design, Setting, and Participants:**

This retrospective cohort study used 2017 to 2021 data from the Optum deidentified Clinformatics Data Mart Database. Each individual in the cohort was categorized into quartiles of OOP costs for a 1-month supply of SGLT2 inhibitor and GLP-1 RA based on their health plan assignment. Data were analyzed from April 2021 to October 2022.

**Exposures:**

OOP cost for SGLT2 inhibitors and GLP-1 RA.

**Main Outcomes and Measures:**

The primary outcome was treatment intensification, defined as a new dispensing (ie, initiation) of either an SGLT2 inhibitor or GLP-1 RA, among patients with T2D previously treated with metformin monotherapy. For each drug class separately, Cox proportional hazards models were used to adjust for demographic, clinical, plan, clinician, and laboratory characteristics to estimate the hazard ratios of treatment intensification comparing the highest vs the lowest quartile of OOP costs.

**Results:**

Our cohort included 80 807 adult patients (mean [SD] age, 72 [9.5] years, 45 129 [55.8%] male; 71 128 [88%] were insured with Medicare Advantage) with T2D and established CVD on metformin monotherapy. Patients were followed for a median (IQR) of 1080 days (528 to 1337). The mean (SD) of OOP costs in the highest vs lowest quartile was $118 [32] vs $25 [12] for GLP-1 RA, and $91 [25] vs $23 [9] for SGLT2 inhibitors. Compared with patients in plans with the lowest quartile (Q1) of OOP costs, patients in plans with the highest quartile (Q4) of costs were less likely to initiate a GLP-1 RA (adjusted HR, 0.87 [95% CI, 0.78 to 0.97]) or an SGLT2 inhibitor (adjusted HR, 0.80 [95% CI, 0.73 to 0.88]). The median (IQR) number of days to initiating a GLP-1 RA was 481 (207-820) days in Q1 and 556 (237-917) days in Q4 of OOP costs and 520 (193-876) days in Q1 vs 685 (309-1017) days in Q4 for SGLT2 inhibitors.

**Conclusions and Relevance:**

In this cohort study of more than 80 000 older adults with T2D and established CVD covered by Medicare Advantage and commercial plans, those in the highest quartile of OOP cost were 13% and 20% less likely to initiate a GLP-1 RA or SGLT2 inhibitor, respectively, when compared with those in the lowest quartile of OOP costs.

## Key Points

**Question:**

What is the association between high out-of-pocket costs and initiation of a sodium-glucose cotransporter 2 (SGLT2) inhibitor or glucagon-like peptide-1 receptor agonist (GLP-1 RA) among adults with type 2 diabetes and established cardiovascular disease?

**Findings:**

This retrospective cohort study examined 80 807 adult patients followed for a median of 1080 days. Compared with patients in plans with the lowest quartile of out-of-pocket costs, patients in plans with the highest quartile of costs were less likely to initiate a GLP-1 RA or an SGLT2 inhibitor.

**Meaning:**

These findings suggest that adults with type 2 diabetes in the highest quartile of OOP costs were 13% and 20% less likely to initiate a GLP-1 RA or SGLT2 inhibitor, respectively, when compared with those in the lowest quartile of OOP costs.

## Teaser

This cohort study assesses the association of high out-of-pocket costs and initiation of a sodium-glucose cotransporter 2 (SGLT2) inhibitor or glucagon-like peptide-1 receptor agonist (GLP-1 RA) among adults who are treated with metformin and have type 2 diabetes and established cardiovascular disease.

## Introduction

Based on evidence from large cardiovascular outcomes trials, the latest professional society guidelines continue to recommend sodium-glucose cotransporter 2 (SGLT2) inhibitors and glucagon-like peptide-1 receptor agonists (GLP-1 RAs) for patients with type 2 diabetes (T2D) and established cardiovascular disease (CVD).^1,2^ Despite these recommendations, data from studies in the clinical setting show that use of these newer drug classes have been paradoxically lower among patients with evidence of, or increased risk factors for, CVD.^3,4,5,6^ Patients with T2D and established CVD tend to be older and have comorbidities, such as hypertension, heart failure, and chronic kidney disease.^7^

High out-of-pocket (OOP) drug costs are identified as a factor that limit initiation of and adherence to prescribed glucose lowering therapies.^8,9,10,11,12^ For example, studies have shown that cost-related nonadherence affects up to 1 in 4 insulin users.^13,14^ Much less is known about the association of high OOP drug costs and use of newer (noninsulin) glucose lowering therapies for patients with T2D.^6^ In prior work from a large integrated health system, we showed that one third of patients newly prescribed an SGLT2 inhibitor or GLP-1 RA did not fill their prescription within 30 days.^15^ However, it is unclear to what extent the underuse of these newer glucose lowering therapies is attributable to cost-related barriers. To address this evidence gap, we assessed the association of OOP costs and initiation of an SGLT2 inhibitor or GLP-1 RA using commercial claims data among adults with T2D and established CVD who are treated with metformin.

## Methods

This cohort study was approved by the University of Pittsburgh institutional review board. Informed consent was waived because this was not human participant research. This report follows Strengthening the Reporting of Observational Studies in Epidemiology (STROBE) reporting guideline.

### Data Source

We conducted a retrospective cohort study using data from 2017 to 2021 from Optum deidentified Clinformatics Data Mart Database (CDM), a longitudinal database that sources deidentified administrative claims for members of commercial and Medicare Advantage plans from the largest health insurer in the US. This database has been used extensively in studies of prescription drug use, adherence and outcomes and includes patient enrollment, physician, facility, and pharmacy claims, and laboratory results.

### Study Population

Adults with T2D with at least 1 claim for dispensed metformin between December 5, 2017, and December 31, 2020, were eligible for our study. We excluded (1) members who had less than 365 days of continuous enrollment prior to their first metformin dispensing; (2) who did not have evidence of established CVD, based on validated *International Statistical Classification of Diseases, Tenth Revision, Clinical Modification *(*ICD-10-CM*) diagnosis and procedure codes and Current Procedural Terminology (CPT) codes during the preindex period^16^ (eTable 1 in Supplement 1); and (3) who had evidence of any second-line glucose lowering drug (eg, SGLT2 inhibitor, GLP-1 RA, thiazolidinedione, dipeptidyl peptidase-4 [DPP-4] inhibitors, sulfonylureas, or insulin) prior to their first metformin dispensing. This ensured that the sample was representative of T2D patients on metformin monotherapy.

### Determining OOP Drug Costs

**Figure 1.**
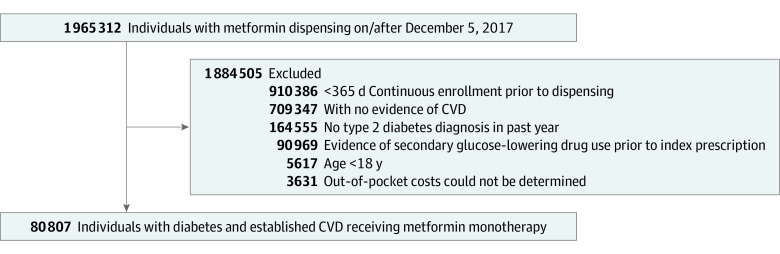
Flow diagram. Abbreviation: CVD, cardiovascular disease.

The Optum CDM database includes detailed information on copays and deductibles for each adjudicated pharmacy claim. Our measure of OOP drug costs summed both copay and deductibles. However, OOP costs cannot be measured for patients who never filled a prescription for an SGLT2 inhibitor or GLP-1 RA. To overcome this limitation, we assigned OOP costs for all members in our cohort by using copay and deductible data from individuals in the Optum CDM database who were not part of our cohort but had the same health plan as our study participants (ie, matched by unique plan identification number). To do this, we first calculated the mean OOP cost within each plan year for all of Optum CDM members not part of our study cohort who had 1 or more fills for an SGLT2 inhibitor or GLP-1 RA (n = 445 873). We then assigned the mean OOP cost to members in our cohort. These costs represent the OOP costs faced by those in the same health insurance plan, allowing us to approximate OOP costs not biased by inclusion of only those who fill prescriptions in the cohort. We scaled all costs to a standard 30-day supply. We excluded members where an OOP cost could not be determined based on all available data (n = 3631) (Figure 1).

### Primary Outcome

Our primary outcome was treatment intensification, defined as a new dispensing (ie, initiation) of either an SGLT2 inhibitor or GLP-1 RA among patients previously treated with metformin monotherapy. We do not use the term treatment intensification to refer to an increase in the dose of a drug.

### Exposures

The primary exposure was OOP cost for SGLT2 inhibitors and GLP-1 RA. In each treatment group separately (SGLT2 inhibitor and GLP-1 RA), we divided members into quartiles based on their mean 30-day OOP cost.

### Covariates

We measured all covariates in the 365 days before their first metformin dispensing during the study time period. Baseline characteristics that were considered to be clinically related to treatment intensification were selected a priori. These included sociodemographic (ie, age, sex, race, geographic region, education, household income), clinical comorbidities (19 variables including heart failure, chronic kidney disease, history of serious hypoglycemic or hyperglycemic events, measures of diabetes complications and specific components of atherosclerotic CVD), insurance type (commercial vs Medicare Advantage), clinician type (endocrinology, cardiology, internal medicine or family practice, or other) of the metformin prescriber, and presence or absence of a hemoglobin A1c (HbA1c) result.

Race was considered in this study because previous studies have demonstrated racial disparities in access to newer glucose lowering medications for Americans living with type 2 diabetes. Race and ethnicity was self-identified at enrollment, and categories included Asian, Black, Hispanic, White, and unknown/missing.

### Statistical Analyses

Summary statistics were reported for the overall cohort as well as for the lowest and highest quartiles of OOP costs for each of SGLT2 inhibitor and GLP-1 RA drug classes. Continuous measures were presented as mean (SD) and categorical measures were presented as No. (%). To depict time until initiation of an SGLT2 inhibitor and GLP-1 RA, we presented cumulative incidence curves (ie, Kaplan-Meier curves) for each drug class separately stratified across the 4 quartiles of OOP costs. For each drug class separately, we used Cox proportional hazards models adjusting for demographics, clinical comorbidities, insurance type, clinician, and laboratory characteristics to estimate the hazards of treatment intensification comparing the highest vs the lowest quartile of OOP costs. Follow-up began on the date of the index metformin dispensing and ended when a member either initiated a target drug of interest or reached the end of the study period (September 30, 2021). Patients were censored if they had a gap in enrollment (plus a grace period of 30 days) or if they disenrolled or died. We constructed 6 Cox models based on the independent variables included as factors of initiation. Model 1 included the quartile of OOP costs only. Model 2 included the quartile of OOP costs and demographic variables. Model 3 included all variables from Model 2 and clinical comorbidities. Model 4 included all variables from Model 3 and insurance type. Model 5 included all variables from Model 4 and included clinician type. Model 6 included all variables from Model 5 as well as the presence (vs absence) of an HbA1c laboratory result.

To improve the rigor of our study, we conducted a falsification test using statin initiation as the outcome of interest. In this test, we wanted to examine whether OOP cost quartiles for SGLT2 inhibitors or GLP-1 RAs were significantly associated with statin initiation. The presence of a strong negative association would suggest the presence of confounding (eg, those with the least OOP costs for SGLT2 inhibitors or GLP-1 RAs are most likely to initiate medications in general compared with those with the highest OOP costs). All analyses were performed using R version 4.2.0 (R Project for Statistical Computing) assuming a Type I error rate α = .05 and no multiplicity adjustments. Data were analyzed from April 2021 to October 2022.

## Results

### Study Participants

Our cohort included 80 807 adult patients (mean [SD] age, 72 [9.5] years, 45 129 [55.8%] male) with T2D and established CVD on metformin monotherapy (Table 1). Most patients (46 135 [57.1%]) had their index metformin dispensed in 2018. This study included 11 555 Black patients (14.3%), 14 420 Hispanic patients (17.8%), and 47 937 White patients (59%). Overall, the presence of clinical comorbidities was high, and 73 724 (91.2%) had hypertension, 71 406 (88.4%) had hyperlipidemia, and 18 264 (22.6%) had heart failure. Most were covered by a Medicare Advantage plan (71 128 [88%]) and had their index metformin prescribed by an internist or family physician (59 759 [74%]). During the 365-day look-back period, 43 191 (53.4%) had an observed HbA1c result, and the mean (SD) HbA1c was 6.79% [1.15%]. Patients were followed for a median (IQR) of 1080 days (528-1337). In the overall cohort, the mean (SD) estimated OOP cost for a 30-day supply of a GLP-1 RA and SGLT2 inhibitor was $69.2 ($38.3) and $54.39 ($28), respectively.

**Table 1.** Baseline Characteristics of the Study Cohort by Lowest and Highest Quartile of Out-of-Pocket Costs for GLP-1 RA or SGLT2 Inhibitors

Characteristics	Total, n = 80 807	OOP costs, No. (%)			
		GLP-1 RA		SGLT2 inhibitors	
		Q1 OOP costs, n = 20 366	Q4 OOP costs, n = 20 158	Q1 OOP costs, n = 20 235	Q4 OOP costs, n = 20 058
Demographics					
Age, mean (SD)	72.22 (9.47)	71.31 (10.28)	73.64 (8.88)	69.91 ( 10.85)	73.28 (8.95)
Sex					
Male	45 129 (55.8)	10 215 (50.2)	12 028 (59.7)	10 405 (51.4)	11 932 (59.5)
Female	35 675 (44.1)	10 151 (49.8)	8130 (40.3)	9827 (48.6)	8126 (40.5)
Division					
East North Central	8563 (10.6)	2431 (11.9)	2727 (13.5)	2401 (11.9)	2875 (14.3)
East South Central	2973 (3.7)	1281 (6.3)	240 (1.2)	1207 (6)	213 (1.1)
Middle Atlantic	6142 (7.6)	747 (3.7)	2650 (13.1)	825 (4.1)	3010 (15)
Mountain	7707 (9.5)	1185 (5.8)	3377 (16.8)	1064 (5.3)	3440 (17.2)
New England	2598 (3.2)	502 (2.5)	622 (3.1)	617 (3)	763 (3.8)
Pacific	12 862 (15.9)	2221 (10.9)	4630 (23)	2314 (11.4)	3719 (18.5)
South Atlantic	20 888 (25.8)	7788 (38.2)	4617 (22.9)	7247 (35.8)	4750 (23.7)
Unknown	115 (0.1)	36 (0.2)	25 (0.1)	37 (0.2)	29 (0.1)
West North Central	3782 (4.7)	913 (4.5)	445 (2.2)	896 (4.4)	380 (1.9)
West South Central	15 177 (18.8)	3262 (16)	825 (4.1)	3627 (17.9)	879 (4.4)
Metformin Fill Year					
2017	18 063 (22.4)	5384 (26.4)	4258 (21.1)	5214 (25.8)	4254 (21.2)
2018	46 135 (57.1)	10 712 (52.6)	12 597 (62.5)	10 150 (50.2)	12 449 (62.1)
2019	8945 (11.1)	2297 (11.3)	1550 (7.7)	2696 (13.3)	1718 (8.6)
2020	7664 (9.5)	1973 (9.7)	1753 (8.7)	2175 (10.7)	1637 (8.2)
Education level					
Less than 12th grade	745 (0.9)	178 (0.9)	62 (0.3)	199 (1)	40 (0.2)
High school diploma	28 593 (35.4)	8483 (41.7)	4808 (23.9)	8391 (41.5)	5073 (25.3)
Less than bachelor’s	40 806 (50.5)	9155 (45)	12 019 (59.6)	9244 (45.7)	11 799 (58.8)
Bachelor’s degree or higher	8157 (10.1)	2082 (10.2)	2695 (13.4)	2023 (10)	2548 (12.7)
Unknown	2506 (3.1)	468 (2.3)	574 (2.8)	378 (1.9)	598 (3)
Household income, $					
<40 000	28 311 (35)	7964 (39.1)	5934 (29.4)	7818 (38.6)	6137 (30.6)
40 000-49 000	8100 (10)	1901 (9.3)	1881 (9.3)	1900 (9.4)	1888 (9.4)
50 000-59 000	8015 (9.9)	1822 (8.9)	1988 (9.9)	1751 (8.7)	2027 (10.1)
60 000-74 000	8824 (10.9)	1934 (9.5)	2447 (12.1)	1907 (9.4)	2388 (11.9)
75 000-99 000	10 171 (12.6)	2256 (11.1)	3015 (15)	2297 (11.4)	2912 (14.5)
≥100 000	11 147 (13.8)	2620 (12.9)	3621 (18)	2759 (13.6)	3404 (17)
Unknown	6239 (7.7)	1869 (9.2)	1272 (6.3)	1803 (8.9)	1302 (6.5)
Race or ethnicity					
Asian	3206 (4)	669 (3.3)	957 (4.7)	698 (3.4)	837 (4.2)
Black	11 555 (14.3)	4335 (21.3)	1600 (7.9)	4091 (20.2)	1841 (9.2)
Hispanic	14 420 (17.8)	3675 (18)	2510 (12.5)	3847 (19)	2404 (12)
White	47 937 (59.3)	10 959 (53.8)	14 105 (70)	10 943 (54.1)	13 997 (69.8)
Unknown	3689 (4.6)	728 (3.6)	986 (4.9)	656 (3.2)	979 (4.9)
Clinical comorbidities					
Heart failure	18 264 (22.6)	4844 (23.8)	4485 (22.2)	4684 (23.1)	4523 (22.5)
Hypertension	73 724 (91.2)	18 831 (92.5)	18 356 (91.1)	18 606 (91.9)	18 423 (91.8)
Hyperlipidemia	71 406 (88.4)	17 946 (88.1)	17 920 (88.9)	17 681 (87.4)	17 890 (89.2)
Chronic kidney disease	19 202 (23.8)	4344 (21.3)	4629 (23)	4098 (20.3)	4350 (21.7)
Serious hypoglycemic events	674 (0.8)	163 (0.8)	173 (0.9)	165 (0.8)	177 (0.9)
Serious hyperglycemic events	748 (0.9)	185 (0.9)	197 (1)	187 (0.9)	205 (1)
Diabetic nephropathy	18 990 (23.5)	3969 (19.5)	4717 (23.4)	3783 (18.7)	4370 (21.8)
Diabetic neuropathy	25 448 (31.5)	6285 (30.9)	5715 (28.4)	6035 (29.8)	5428 (27.1)
Diabetic retinopathy	8897 (11)	1889 (9.3)	2035 (10.1)	1789 (8.8)	1813 (9)
Foot ulcers	19 038 (23.6)	4294 (21.1)	4186 (20.8)	4197 (20.7)	3836 (19.1)
End stage kidney disease	875 (1.1)	225 (1.1)	238 (1.2)	221 (1.1)	243 (1.2)
Obesity	22 530 (27.9)	5926 (29.1)	5258 (26.1)	6180 (30.5)	5341 (26.6)
Smoker	28 252 (35)	7282 (35.8)	6947 (34.5)	7349 (36.3)	7131 (35.6)
Unstable angina	3262 (4)	859 (4.2)	874 (4.3)	889 (4.4)	913 (4.6)
Peripheral artery disease	37 168 (46)	8766 (43)	8646 (42.9)	8725 (43.1)	8207 (40.9)
Myocardial infarction	20 789 (25.7)	5258 (25.8)	5376 (26.7)	5448 (26.9)	5445 (27.1)
Coronary artery bypass graft	13 354 (16.5)	3292 (16.2)	3671 (18.2)	3085 (15.2)	3686 (18.4)
Percutaneous coronary intervention	19 239 (23.8)	4974 (24.4)	5065 (25.1)	4969 (24.6)	5162 (25.7)
Stroke	17 513 (21.7)	4819 (23.7)	4433 (22)	4746 (23.5)	4573 (22.8)
Insurance type					
Commercial	9679 (12)	2994 (14.7)	1961 (9.7)	4616 (22.8)	2056 (10.3)
Medicare Advantage	71 128 (88)	17 372 (85.3)	18 197 (90.3)	15 619 (77.2)	18 002 (89.7)
Clinician specialty					
Family Practice/Internal Medicine	59 759 (74)	14 874 (73)	14 821 (73.5)	14 686 (72.6)	14 708 (73.3)
Endocrinology	1624 (2)	420 (2.1)	468 (2.3)	420 (2.1)	468 (2.3)
Cardiology	1338 (1.7)	348 (1.7)	400 (2)	360 (1.8)	422 (2.1)
Unknown	7945 (9.8)	2044 (10)	1999 (9.9)	1952 (9.6)	1966 (9.8)
Other	10 141 (12.5)	2680 (13.2)	2470 (12.3)	2817 (13.9)	2494 (12.4)
HBA1C result					
Present HBA1C result	43 191 (53.4)	9083 (44.6)	11 608 (57.6)	9194 (45.4)	11 190 (55.8)
HBA1C (%)	6.79 (1.15)	6.74 (1.13)	6.76 (1.1)	6.78 (1.19)	6.79 (1.14)
Average 30-d OOP cost					
GLP-1 RA, mean (SD), $	69.2 (38.29)	25.08 (12.33)	118.43 (32.11)	27.95 (20.32)	114.31 (33.35)
SGLT2 inhibitor, mean (SD), $	54.39 (28)	25.49 (17)	86.89 (24.05)	23.19 (9.42)	90.73 (24.71)

Abbreviations: GLP-1 RA, glucagon-like peptide 1 receptor agonist; OOP, out-of-pocket; Q1, lowest quartile; Q4, highest quartile; SGLT2, sodium-glucose cotransporter 2.

Among those in the GLP-1 RA cohort, those in the highest quartile of OOP costs were slightly older (mean age 73.6 vs 71.3), more likely to be male (59.7% vs 50.2%), more likely to be White (70% vs 53.8%), and had a higher proportion with diabetic nephropathy (23.4% vs 19.5%), all when compared against those in the lowest quartile of OOP costs (Table 1). In addition, those in the highest quartile of OOP costs also had higher income categories and were more likely to be covered by a Medicare Advantage plan (18 197 [90.3%] vs 17 372 [85.3%]). Patients in the lowest quartile faced mean (SD) OOPs cost of $25.08 ($12.33) vs $118.43 ($32.11) in the highest quartile.

For those in the SGLT2 inhibitor cohort, those in the highest quartile of OOP costs were similarly older (mean [SD] age, 73.3 [9.0] years vs 69.9 [10.9] years), more likely to be male (11 932 [59.5%] vs 10 405 [51.4%]), more likely to be White (13 997 [69.8%] vs 10 943 [54.1%]), and had a higher proportion with diabetic nephropathy (4370 [21.8%] vs 3783 [18.7%]), all when compared against those in the lowest quartile of OOP costs (Table 1). Patients in the lowest faced mean OOP costs of $23.19 [$9.42] vs $90.73 [$24.71] in the highest quartile.

### Likelihood of Treatment Intensification by Quartile of OOP Costs

**Figure 2.**
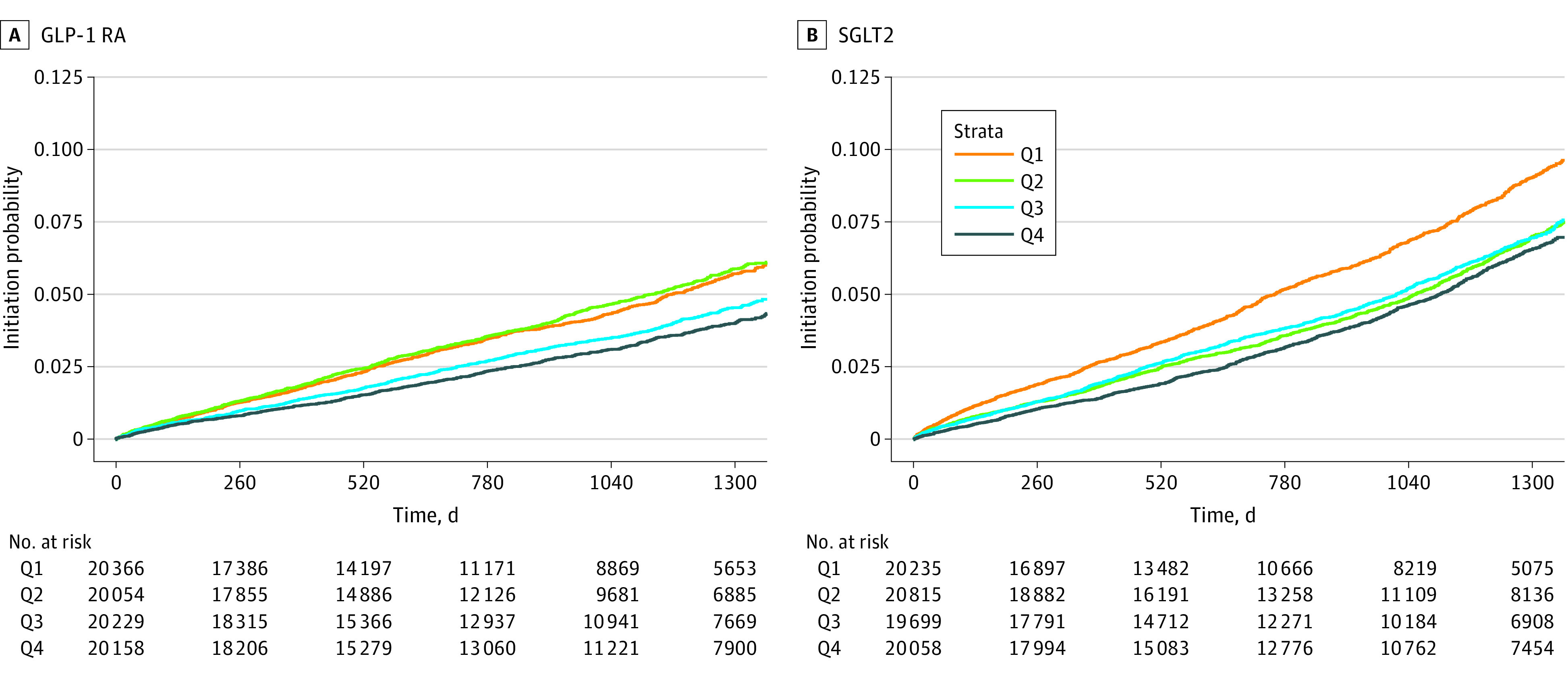
Cumulative Incidence Curve for GLP-1 RA and SGLTI Initiation Stratified by Q1 Through Q4 of OOP Costs Abbreviations: GLP-1 RA, glucagon-like peptide 1 receptor agonist; OOP, out-of-pocket; Q, quartile; SGLTI, sodium-glucose cotransporter 2 inhibitor.

Compared with those in the plans with the lowest quartile of OOP costs, individuals in the highest quartile were least likely to initiate a GLP-1 RA, with an unadjusted HR of 0.70 (95% CI, 0.63-0.78) (Table 2 and Figure 2). In the model that adjusted for all covariates, the HR attenuated to 0.87 (95% CI, 0.78-0.97) but remained statistically significant.

**Table 2.** Hazard Ratio of Initiating a GLP-1 RA or SGLT2 Inhibitor Comparing the Highest vs the Lowest Quartile of OOP Costs

Model No.	Description of model adjustments	Initiation hazard ratio (95% CI)^a^	
		GLP-1 RA	SGLT2 inhibitor
1	Quartile of OOP costs only	0.70 (0.63 to 0.78)	0.68 (0.62 to 0.74)
2	Model 1+demographics^b^	0.86 (0.77 to 0.96)	0.78 (0.71 to 0.86)
3	Model 2+comorbidities^c^	0.86 (0.77 to 0.96)	0.78 (0.71 to 0.85)
4	Model 3+insurance type	0.86 (0.77 to 0.97)	0.80 (0.73 to 0.88)
5	Model 4+clinician type	0.86 (0.77 to 0.97)	0.80 (0.73 to 0.88)
6	Model 5+has HbA1c lab result	0.87 (0.78 to 0.97)	0.80 (0.73 to 0.88)

Abbreviations: GLP-1 RA, glucagon-like peptide 1 receptor agonist; HbA1c, hemoglobin A1C; OOP, out-of-pocket; Q1, lowest quartile; Q4, highest quartile; SGLT2, sodium-glucose cotransporter 2.

^a^ Q4 vs Q1 OOP costs.

^b^ Age, gender, division, metformin fill year, education level, household income range, race.

^c^ Heart failure, hypertension, hyperlipidemia, chronic kidney disease, serious hypoglycemic events, serious hyperglycemic events, diabetic nephropathy, diabetic neuropathy, diabetic retinopathy, foot ulcers, end state renal disease, obesity, smoking status, unstable angina, peripheral artery disease, myocardial infarction, coronary artery bypass graft, percutaneous coronary intervention, stroke.

Similarly, for SGLT2 inhibitors, those in the highest vs the lowest quartile of OOP costs had an unadjusted HR of 0.68 (95% CI, 0.62-0.74) for SGLT2 inhibitor initiation (Table 2 and Figure 2). In the most comprehensive model, adjusting for demographic, comorbidities, insurance type and presence of HbA1c, the HR was 0.80 (95% CI, 0.73-0.88). Full model outputs are shown in eTable 2 to eTable 7 in Supplement 1.

**Figure 3.**
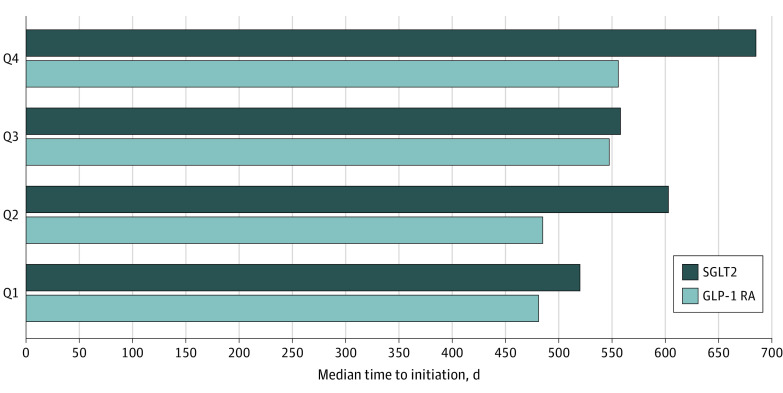
Median Number of Days to Initiating a GLP-1 RA or SGLTI, by Quartile of OOP Costs Abbreviations: GLP-1 RA, glucagon-like peptide 1 receptor agonist; OOP, out-of-pocket; Q, quartile; SGLTI, sodium-glucose cotransporter 2 inhibitor.

The median (IQR) number of days between the index metformin dispensing and initiation of a GLP-1 RA was 481 (207-820) days, 485 (210-874) days, 548 (234-889) days, and 556 (237-917) days for Q1, Q2, Q3, and Q4 of OOP costs, respectively (Figure 3). The median number of days between the index metformin dispensing and initiation of an SGLT2 inhibitor was 520 (193-876) days, 603 (274-1033) days, 558 (263-966) days, and 685 (309-1017) days for Q1, Q2, Q3, and Q4 of OOP costs, respectively.

### Statin Falsification Test

We compared the hazards of statin initiation among the quartiles of OOP costs for GLP-1 RAs and SGLT2 inhibitors. Individuals in the highest quartile of OOP costs had similar (but slightly higher) likelihood of statin initiation compared with those in the lowest quartile of OOP costs (adjusted HR, 1.08; 95% CI, 1.01 to 1.16 for Q4 vs Q1 of OOP costs for GLP-1 RA and adjusted HR 1.10, 95% CI 1.03 to 1.18 for Q4 vs Q1 of OOP costs for SGLT2 inhibitors) (eFigure 1 and 2 and eTable 8 in Supplement 1).

## Discussion

In this nationwide cohort study of more than 80 000 older adults with T2D and CVD, we found that those in the highest quartile of OOP costs were 13% and 20% less likely to initiate a GLP-1 RA or SGLT2 inhibitor, respectively, when compared with those in the lowest quartile of OOP costs. For this high risk group, observed utilization rates for these 2 classes of medications were very low. In addition, patients in the highest OOP 2 category experienced a median delay in treatment intensification of 75 days for GLP-1 RAs and 165 days for SGLT2 inhibitors when compared to patients in the lowest OOP cost category. To put this in a clinical context, this means that among older adults with T2D and established CVD, patients in the highest OOP quartile received intensification to guideline recommended therapies a median of 3 to 6 months later than patients in the lowest OOP cost quartile. Such delays may reduce the potential impact of these 2 classes of medications on important clinical outcomes, such as myocardial infarction, stroke, hospitalization for heart failure, and worsening of renal function; thereby resulting in higher downstream costs associated with treating these complications.

Prior studies examining trends in the use of these newer classes of glucose lowering drugs uncovered a treatment paradox where those with the most comorbidities (and who likely have the strongest clinical indications for using selected agents within these classes for cardiovascular risk reduction) were least likely to start 1 of these medications.^4,6^ Our study adds to this literature by using more contemporary data and focusing more specifically on OOP costs. Our findings suggest that high drug costs may independently estimate the likelihood of treatment initiation with an SGLT2 inhibitor or GLP-1 RA, irrespective of a patient’s age, race, degree of glucose control, and the presence of preexisting CVD or other clinical comorbidities. Although we believe this is the first study to demonstrate the association of high OOP costs and treatment initiation for these medications, prior literature has documented this association in patients with both diabetes^8,9,12^ and multiple other conditions.^17,18,19^

There has been recent policy action to address high prescription costs, especially among Medicare beneficiaries. Recently, the president signed the Inflation Reduction Act of 2022, which institutes a $35 copay cap for insulin among Medicare beneficiaries.^20,21^ The copay cap does not have any effect on those with commercial insurance, the uninsured,^22^ or those receiving SGLT2 inhibitors or GLP-1 RAs, which are increasingly recommended as first line agents for patients with diabetes and preexisting cardiovascular or renal disease. However, the bill does redesign the Medicare Part D benefit and institutes a $2000 OOP cap on copayments, which would have a beneficial effect on beneficiaries who take these medications.^21^ Strengths of our study include its use of a large administrative claims database, large sample size, and focus on a specific population of patients with strong indications for use of newer glucose lowering medications (ie, those with established or preexisting CVD).

### Limitations

This study had limitations. First, our results were drawn from 1 national health insurance company with most patients covered under Medicare Advantage. Therefore, our results may not be generalizable to patients covered by other commercial plans, Medicaid beneficiaries, or individuals with Medicare fee-for-service. Future studies should consider the impact of OOP costs on medication initiation among patients covered by these plans. Second, we did not have access to formulary data which precluded a precise calculation of each member’s exact OOP cost for SGLT2 inhibitors or GLP-1 RA. However, we did estimate OOP costs using data drawn from other Optum CDM members with the same unique plan identification number and year of index metformin dispensing as members in our cohort, which we believe addresses a shortcoming of trying to measure OOP costs for those who do not fill a medication. Third, Optum CDM claims did not include information on samples or prescriptions paid with cash or reimbursed through secondary payers, which may have led to outcome misclassification. Notably, our results would not likely be biased by differential use of manufacturer-sponsored drug coupons since the use of these types of discounts are not allowed in Medicare plans. Fourth, our results may not apply to all patients since SGLT2 inhibitors and GLP-1 RAs are increasingly being used as front-line agents in T2D, while our cohort entry criteria required prior use of metformin. Fifth, our results may be subject to residual confounding due to unmeasured factors, although our use of a statin initiation falsification test is a strength and suggests that bias related to who enrolls in higher OOP plans does not explain our findings on SGLT2 inhibitor and GLP-1 RA initiation. Important areas for future research include whether overall OOP costs are associated with the use of these therapies and whether the findings we describe here also apply to medication adherence.

## Conclusions

In this cohort study using data from more than 80 000 patients with T2D and established CVD, those with the greatest cost-sharing for SGLT2 inhibitors and GLP-1 RA had a significantly lower likelihood of treatment intensification to a drug class with proven cardiovascular benefits. The median duration of delay comparing the highest vs the lowest quartile of OOP costs ranged from 3 to 6 months. Future studies should examine whether these differences in prescribing translate into meaningful differences in outcomes that are meaningful to patients in clinical settings.

## Notes

Supplement 1. **eTable 1.** *ICD-9-CM*, *ICD-10-CM* Diagnostic and Procedure Codes Used to Define Prevalent Atherosclerotic Cardiovascular Disease **eTable 2.** Full Cox Proportional Hazards Model Output, Model #1 **eTable 3.** Full Cox Proportional Hazards Model Output, Model #2 **eTable 4.** Full Cox Proportional Hazards Model Output, Model #3 **eTable 5.** Full Cox Proportional Hazards Model Output, Model #4 **eTable 6.** Full Cox Proportional Hazards Model Output, Model #5 **eTable 7.** Full Cox Proportional Hazards Model Output, Model #6 **eFigure 1.** Cumulative Incidence Curve for Statin Initiation Stratified by Q1 Through Q4 of OOP Costs for GLP-1 RA **eFigure 2.** Cumulative Incidence Curve for Statin Initiation Stratified by Q1 Through Q4 of OOP Costs for SGLT2 Inhibitors **eTable 8.** Hazard Ratio of Initiating a Statin Comparing the Highest vs the Lowest Quartile of OOP Costs for GLP-1 Receptor Agonists or SGLT2 Inhibitors

Supplement 2.
Data Sharing Statement
