# Supplementary material for: Evaluation of Out-of-Pocket Costs and Treatment Intensification With an SGLT2 Inhibitor or GLP-1 RA in Patients With Type 2 Diabetes and Cardiovascular Disease
Source: JAMA Netw Open. 2023 Jun 12;6(6):e2317886. doi: 10.1001/jamanetworkopen.2023.17886 (PMC10261995; doi:10.1001/jamanetworkopen.2023.17886)
Supplement: Supplement 1. — eTable 1. ICD-9-CM, ICD-10-CM Diagnostic and Procedure Codes Used to Define Prevalent Atherosclerotic Cardiovascular Disease eTable 2. Full Cox Proportional Hazards Model Output, Model #1 eTable 3. Full Cox Proportional Hazards Model Output, Model #2 eTable 4. Full Cox Proportional Hazards Model Output, Model #3 eTable 5. Full Cox Proportional Hazards Model Output, Model #4 eTable 6. Full Cox Proportional Hazards Model Output, Model #5 eTable 7. Full Cox Proportional Hazards Model Output, Model #6 eFigure 1. Cumulative Incidence Curve for Statin Initiation Stratified by Q1 Through Q4 of OOP Costs for GLP-1 RA eFigure 2. Cumulative Incidence Curve for Statin Initiation Stratified by Q1 Through Q4 of OOP Costs for SGLT2 Inhibitors eTable 8. Hazard Ratio of Initiating a Statin Comparing the Highest vs the Lowest Quartile of OOP Costs for GLP-1 Receptor Agonists or SGLT2 Inhibitors [file jamanetwopen-e2317886-s001.pdf]

Supplemental Online Content

Luo J, Feldman R, Callaway Kim K, et al. Evaluation of out-of-pocket costs and treatment intensification with a sodium glucose cotransporter 2 inhibitor or glucagon-like peptide-1 receptor agonist in patients with type 2 diabetes and cardiovascular disease. *JAMA Netw Open*. 2023;6(6):e2317886. doi:10.1001/jamanetworkopen.2023.17886

eTable 1. *ICD-9-CM*, *ICD-10-CM* Diagnostic and Procedure Codes Used to Define Prevalent Atherosclerotic Cardiovascular Disease

eTable 2. Full Cox Proportional Hazards Model Output, Model #1

eTable 3. Full Cox Proportional Hazards Model Output, Model #2

eTable 4. Full Cox Proportional Hazards Model Output, Model #3

eTable 5. Full Cox Proportional Hazards Model Output, Model #4

eTable 6. Full Cox Proportional Hazards Model Output, Model #5

eTable 7. Full Cox Proportional Hazards Model Output, Model #6

eFigure 1. Cumulative Incidence Curve for Statin Initiation Stratified by Q1 Through Q4 of OOP Costs for GLP-1 RA

eFigure 2. Cumulative Incidence Curve for Statin Initiation Stratified by Q1 Through Q4 of OOP Costs for SGLT2 Inhibitors

eTable 8. Hazard Ratio of Initiating a Statin Comparing the Highest vs the Lowest Quartile of OOP Costs for GLP-1 Receptor Agonists or SGLT2 Inhibitors

This supplemental material has been provided by the authors to give readers additional information about their work.

© 2023 Luo J et al. *JAMA Network Open.*

eTable 1. *ICD-9-CM, ICD-10-CM* diagnostic and procedure codes used to define prevalent atherosclerotic cardiovascular disease (ASCVD).

Diagnosis	Codes	Description	Codes	Validation Study
Stroke/TIA (Cerebrovascular Disease)	9	Regex	“^43[0-8]”	
	10		“^I63”, “^G45[.]9”	
Unstable Angina	9	Regex	411.1	
	10		I20.0	
Peripheral Artery Disease	9	Exact	“250.70”	
	10	Regex	“^E11[.]5”, “^E13[.]5"	
CAD/CABG/PCI	9 or 10	Regex	Codes for myocardial infarction:<br>ICD-9 diagnosis codes<br>'41000', '41001', '41002', '41010', '41011', '41012', '41020', '41021', '41022', '41030', '41031', '41032', '41040', '41041', '41042', '41050', '41051', '41052', '41060', '41061', '41062', '41070', '41071', '41072', '41080', '41081', '41082', '41090', '41091', '41092', '412'<br>-or-<br>ICD-10 diagnosis codes<br>'I2101', 'I2102', 'I2109', 'I2111', 'I2119', 'I2121', 'I2129', 'I213', 'I214', 'I220', 'I221', 'I222', 'I228', 'I229', 'I252'<br><br>Codes for CABG:<br>ICD-9 diagnosis code ‘V4581’<br>or<br>ICD-10 diagnosis code 'Z951'<br>or<br>ICD-9 procedure codes '3610', '3611', '3612', '3613', '3614', '3615', '3616', '3617', '3619'<br>or<br>ICD-10 procedure codes '0210093', '0210098', '0210099', '021009C', '021009F', '021009W', '02100A3', '02100A8', '02100A9', '02100AC', '02100AF', '02100AW', '02100J3', '02100J8', '02100J9', '02100JC', '02100JF', '02100JW', '02100K3', '02100K8', '02100K9', '02100KC', '02100KF', '02100KW', '02100Z3', '02100Z8', '02100Z9', '02100ZC', '02100ZF', '0210344', '02103D4', '0210444', '0210493', '0210498', '0210499', '021049C', '021049F', '021049W', '02104A3', '02104A8', '02104A9', '02104AC', '02104AF', '02104AW', '02104D4', '02104J3', '02104J8', '02104J9', '02104JC', '02104JF', '02104JW', '02104K3',	Fishman et al., Contemporary Clinical Communications, 2018

© 2023 Luo J et al. *JAMA Network Open.*

			'02104K8', '02104K9', '02104KC', '02104KF', '02104KW', '02104Z3', '02104Z8', '02104Z9', '02104ZC', '02104ZF', '0211093', '0211098', '0211099', '021109C', '021109F', '021109W', '02110A3', '02110A8', '02110A9', '02110AC', '02110AF', '02110AW', '02110J3', '02110J8', '02110J9', '02110JC', '02110JF', '02110JW', '02110K3', '02110K8', '02110K9', '02110KC', '02110KF', '02110KW', '02110Z3', '02110Z8', '02110Z9', '02110ZC', '02110ZF', '0211344', '02113D4', '0211444', '0211493', '0211498', '0211499', '021149C', '021149F', '021149W', '02114A3', '02114A8', '02114A9', '02114AC', '02114AF', '02114AW', '02114D4', '02114J3', '02114J8', '02114J9', '02114JC', '02114JF', '02114JW', '02114K3', '02114K8', '02114K9', '02114KC', '02114KF', '02114KW', '02114Z3', '02114Z8', '02114Z9', '02114ZC', '02114ZF', '0212093', '0212098', '0212099', '021209C', '021209F', '021209W', '02120A3', '02120A8', '02120A9', '02120AC', '02120AF', '02120AW', '02120J3', '02120J8', '02120J9', '02120JC', '02120JF', '02120JW', '02120K3', '02120K8', '02120K9', '02120KC', '02120KF', '02120KW', '02120Z3', '02120Z8', '02120Z9', '02120ZC', '02120ZF', '0212344', '02123D4', '0212444', '0212493', '0212498', '0212499', '021249C', '021249F', '021249W', '02124A3', '02124A8', '02124A9', '02124AC', '02124AF', '02124AW', '02124D4', '02124J3', '02124J8', '02124J9', '02124JC', '02124JF', '02124JW', '02124K3', '02124K8', '02124K9', '02124KC', '02124KF', '02124KW', '02124Z3', '02124Z8', '02124Z9', '02124ZC', '02124ZF', '0213093', '0213098', '0213099', '021309C', '021309F', '021309W', '02130A3', '02130A8', '02130A9', '02130AC', '02130AF', '02130AW', '02130J3', '02130J8', '02130J9', '02130JC', '02130JF', '02130JW', '02130K3', '02130K8', '02130K9', '02130KC', '02130KF', '02130KW', '02130Z3', '02130Z8', '02130Z9', '02130ZC', '02130ZF', '0213344', '02133D4', '0213444', '0213493', '0213498', '0213499', '021349C', '021349F', '021349W', '02134A3', '02134A8', '02134A9', '02134AC', '02134AF', '02134AW', '02134D4', '02134J3', '02134J8', '02134J9', '02134JC', '02134JF',<br><br>'02134JW', '02134K3', '02134K8', '02134K9', '02134KC', '02134KF', '02134KW', '02134Z3', '02134Z8', '02134Z9', '02134ZC', '02134ZF'	

© 2023 Luo J et al. *JAMA Network Open.*

			or<br>CPT/HCPCS codes '33510', '33511', '33512', '33513', '33514', '33516', '33517', '33518', '33519', '33521', '33522', '33523', '33533', '33534', '33535', '33536'<br><br>Codes for PCI:<br>ICD-9 Diagnosis code V4582<br>or<br>ICD-10 Diagnosis code ‘Z955’,’Z9861’<br>or<br>ICD-9 Procedure code '0066', '1755', '3601', '3602', '3605', '3606', '3607', '3609'<br>or<br>ICD-10 Procedure codes '0270046', '027004Z', '02700D6', '02700DZ', '02700T6', '02700TZ', '02700Z6', '02700ZZ', '0270346', '027034Z', '02703D6', '02703DZ', '02703T6', '02703TZ', '02703Z6', '02703ZZ', '0270446', '027044Z', '02704D6', '02704DZ', '02704T6', '02704TZ', '02704Z6', '02704ZZ', '0271046', '027104Z', '02710D6', '02710DZ', '02710T6', '02710TZ', '02710Z6', '02710ZZ', '0271346', '027134Z', '02713D6', '02713DZ', '02713T6', '02713TZ', '02713Z6', '02713ZZ', '0271446', '027144Z', '02714D6', '02714DZ', '02714T6', '02714TZ', '02714Z6', '02714ZZ', '0272046', '027204Z', '02720D6', '02720DZ', '02720T6', '02720TZ', '02720Z6', '02720ZZ', '0272346', '027234Z', '02723D6', '02723DZ', '02723T6', '02723TZ', '02723Z6', '02723ZZ', '0272446', '027244Z', '02724D6', '02724DZ', '02724T6', '02724TZ', '02724Z6', '02724ZZ', '0273046', '027304Z', '02730D6', '02730DZ', '02730T6', '02730TZ', '02730Z6', '02730ZZ', '0273346', '027334Z', '02733D6', '02733DZ', '02733T6', '02733TZ', '02733Z6', '02733ZZ', '0273446', '027344Z', '02734D6', '02734DZ', '02734T6', '02734TZ', '02734Z6', '02734ZZ', '02C03ZZ', '02C04ZZ', '02C13ZZ', '02C14ZZ', '02C23ZZ', '02C24ZZ', '02C33ZZ', '02C34ZZ', 'X2C0361', 'X2C1361'<br>or<br>CPT/HCPCS codes '92920', '92921', '92924', '92925', '92928', '92929', '92933', '92934', '92937', '92938', '92941', '92943', '92944', '92980', '92981', '92982', '92984', '92995', '92996', 'C9600', 'C9601', 'C9602', 'C9603', 'C9604', 'C9605', 'C9606', 'C9607', 'C9608', 'G0290', 'G0291'	

© 2023 Luo J et al. *JAMA Network Open.*

eTable 2. Full cox proportional hazards model output, Model #1

	GLP-1a initiation				SGLT2i initiation			
**Variable**	**Hazard ratio**	**95% Conf. Interval**	**P-value**	**Omnibus P-value**	**Hazard ratio**	**95% Conf. Interval**	**P-value**	**Omnibus P-value**
Quartile of OOP Costs				<0.001				<0.001
1	Ref.	Ref.	Ref.		Ref.	Ref.	Ref.	
2	1.032	(0.936, 1.138)	0.524		0.74	(0.681, 0.804)	<0.001	
3	0.79	(0.713, 0.876)	<0.001		0.754	(0.692, 0.82)	<0.001	
4	0.696	(0.626, 0.775)	<0.001		0.676	(0.62, 0.737)	<0.001	

OOP = Out-of-pocket; Ref. = Reference

© 2023 Luo J et al. *JAMA Network Open.*

eTable 3. Full cox proportional hazards model output, Model #2

	GLP-1a initiation				SGLT2i initiation			
**Variable**	**Hazard ratio**	**95% Conf. Interval**	**P-value**	**Omnibus P-value**	**Hazard ratio**	**95% Conf. Interval**	**P-value**	**Omnibus P-value**
Quartile of OOP Costs				0.003				<0.001
1	Ref.	Ref.	Ref.		Ref.	Ref.	Ref.	
2	1.049	(0.95, 1.159)	0.346		0.91	(0.836, 0.992)	0.032	
3	0.932	(0.838, 1.038)	0.199		0.866	(0.793, 0.946)	0.001	
4	0.859	(0.768, 0.96)	0.008		0.782	(0.713, 0.857)	<0.001	
Age in years	0.937	(0.934, 0.94)	<0.001		0.954	(0.951, 0.957)	<0.001	
Sex				<0.001				<0.001
Male	Ref.	Ref.	Ref.		Ref.	Ref.	Ref.	
Female	1.203	(1.116, 1.297)	<0.001		0.723	(0.677, 0.772)	<0.001	
Unknown	0	(0, 2.2e+280)	0.981		0	(0, 1.7e+246)	0.978	
Geographical Division				<0.001				<0.001
East North Central	Ref.	Ref.	Ref.		Ref.	Ref.	Ref.	
East South Central	1.249	(1.03, 1.516)	0.024		1.023	(0.858, 1.221)	0.798	
Middle Atlantic	0.901	(0.747, 1.086)	0.274		0.996	(0.859, 1.156)	0.962	
Mountain	0.831	(0.703, 0.983)	0.03		0.807	(0.702, 0.928)	0.003	
New England	0.888	(0.697, 1.13)	0.334		1.06	(0.881, 1.276)	0.535	
Pacific	0.821	(0.703, 0.958)	0.013		0.914	(0.809, 1.032)	0.148	
South Atlantic	0.975	(0.855, 1.112)	0.711		0.936	(0.838, 1.045)	0.239	
Unknown	0.699	(0.224, 2.181)	0.538		0.613	(0.229, 1.642)	0.33	
West North Central	1.187	(0.996, 1.416)	0.056		1.097	(0.941, 1.28)	0.236	
West South Central	0.956	(0.83, 1.102)	0.537		0.794	(0.703, 0.897)	<0.001	
Metformin fill year				<0.001				<0.001
2017	Ref.	Ref.	Ref.		Ref.	Ref.	Ref.	
2018	1.203	(1.096, 1.321)	<0.001		1.196	(1.104, 1.295)	<0.001	
2019	1.58	(1.38, 1.809)	<0.001		2.111	(1.885, 2.365)	<0.001	
2020	2.102	(1.795, 2.461)	<0.001		3.36	(2.956, 3.818)	<0.001	
Education				0.493				0.206
<12th grade	Ref.	Ref.	Ref.		Ref.	Ref.	Ref.	
High school diploma	1.122	(0.709, 1.775)	0.623		0.849	(0.61, 1.182)	0.332	
<Bachelor's degree	1.137	(0.717, 1.801)	0.585		0.867	(0.622, 1.209)	0.399	
≥Bachelor's degree	1.091	(0.677, 1.758)	0.722		0.965	(0.682, 1.364)	0.838	
Unknown/missing	0.804	(0.439, 1.471)	0.479		0.998	(0.631, 1.58)	0.994	
Household income				<0.001				0.002
<$40K	Ref.	Ref.	Ref.		Ref.	Ref.	Ref.	
$40K-$49K	1.133	(0.995, 1.289)	0.059		1.069	(0.957, 1.195)	0.238	
$50K-$59K	0.956	(0.831, 1.101)	0.535		1.124	(1.006, 1.255)	0.038	
$60K-$74K	1.105	(0.971, 1.258)	0.13		1.058	(0.949, 1.18)	0.309	
$75K-$99K	1.217	(1.079, 1.373)	0.001		1.164	(1.052, 1.289)	0.003	
$100K+	1.236	(1.091, 1.401)	0.001		1.127	(1.015, 1.253)	0.026	
Unknown/missing	0.78	(0.621, 0.979)	0.032		0.811	(0.672, 0.98)	0.03	
Race				<0.001				0.024
White	Ref.	Ref.	Ref.		Ref.	Ref.	Ref.	
Black	0.817	(0.73, 0.914)	<0.001		0.919	(0.834, 1.013)	0.089	
Asian	0.38	(0.282, 0.51)	<0.001		0.812	(0.686, 0.962)	0.016	
Hispanic	0.777	(0.694, 0.869)	<0.001		0.91	(0.831, 0.998)	0.045	

© 2023 Luo J et al. *JAMA Network Open.*

Unknown/missing	1.295	(1.011, 1.657)	0.04		1.059	(0.847, 1.324)	0.615	

OOP = Out-of-pocket; Ref. = Reference

© 2023 Luo J et al. *JAMA Network Open.*

eTable 4. Full cox proportional hazards model output, Model #3

	GLP-1a initiation				SGLT2i initiation			
**Variable**	**Hazard ratio**	**95% Conf. Interval**	**P-value**	**Omnibus P-value**	**Hazard ratio**	**95% Conf. Interval**	**P-value**	**Omnibus P-value**
Quartile of OOP Costs				0.002				<0.001
1	Ref.	Ref.	Ref.		Ref.	Ref.	Ref.	
2	1.048	(0.948, 1.157)	0.36		0.914	(0.839, 0.995)	0.039	
3	0.917	(0.824, 1.021)	0.114		0.871	(0.797, 0.952)	0.002	
4	0.858	(0.767, 0.96)	0.007		0.778	(0.71, 0.853)	<0.001	
Age in years	0.939	(0.935, 0.942)	<0.001		0.953	(0.95, 0.956)	<0.001	
Sex				<0.001				<0.001
Male	Ref.	Ref.	Ref.		Ref.	Ref.	Ref.	
Female	1.195	(1.106, 1.291)	<0.001		0.771	(0.721, 0.825)	<0.001	
Unknown	0.001	(0, 2.4e+286)	0.982		0	(0, 2.9e+247)	0.979	
Geographical Division				<0.001				<0.001
East North Central	Ref.	Ref.	Ref.		Ref.	Ref.	Ref.	
East South Central	1.249	(1.029, 1.516)	0.025		1.011	(0.847, 1.207)	0.904	
Middle Atlantic	0.924	(0.766, 1.115)	0.41		1.013	(0.872, 1.175)	0.87	
Mountain	0.809	(0.684, 0.958)	0.014		0.835	(0.726, 0.96)	0.011	
New England	0.907	(0.712, 1.155)	0.427		1.095	(0.91, 1.319)	0.335	
Pacific	0.801	(0.685, 0.938)	0.006		0.96	(0.849, 1.087)	0.522	
South Atlantic	0.973	(0.853, 1.11)	0.679		0.949	(0.85, 1.06)	0.351	
Unknown	0.696	(0.223, 2.173)	0.533		0.606	(0.226, 1.624)	0.319	
West North Central	1.177	(0.987, 1.404)	0.069		1.104	(0.947, 1.288)	0.206	
West South Central	0.925	(0.801, 1.067)	0.285		0.817	(0.722, 0.924)	0.001	
Metformin fill year				<0.001				<0.001
2017	Ref.	Ref.	Ref.		Ref.	Ref.	Ref.	
2018	1.196	(1.089, 1.313)	<0.001		1.2	(1.108, 1.3)	<0.001	
2019	1.579	(1.378, 1.809)	<0.001		2.128	(1.899, 2.384)	<0.001	
2020	2.098	(1.79, 2.458)	<0.001		3.428	(3.014, 3.897)	<0.001	
Education				0.493				0.127
<12th grade	Ref.	Ref.	Ref.		Ref.	Ref.	Ref.	
High school diploma	1.123	(0.71, 1.776)	0.619		0.831	(0.597, 1.157)	0.273	
<Bachelor's degree	1.149	(0.725, 1.82)	0.555		0.853	(0.612, 1.19)	0.35	
≥Bachelor's degree	1.12	(0.695, 1.805)	0.641		0.958	(0.678, 1.355)	0.81	
Unknown/missing	0.808	(0.441, 1.48)	0.49		0.99	(0.625, 1.566)	0.965	
Household income				<0.001				0.005
<$40K	Ref.	Ref.	Ref.		Ref.	Ref.	Ref.	
$40K-$49K	1.148	(1.008, 1.306)	0.037		1.073	(0.96, 1.199)	0.217	
$50K-$59K	0.978	(0.849, 1.126)	0.756		1.128	(1.01, 1.26)	0.033	
$60K-$74K	1.123	(0.986, 1.279)	0.08		1.059	(0.949, 1.182)	0.303	
$75K-$99K	1.238	(1.097, 1.398)	0.001		1.166	(1.053, 1.292)	0.003	
$100K+	1.275	(1.124, 1.446)	<0.001		1.124	(1.011, 1.251)	0.031	
Unknown/missing	0.801	(0.638, 1.005)	0.055		0.833	(0.69, 1.006)	0.057	
Race				<0.001				0.16

© 2023 Luo J et al. *JAMA Network Open.*

White	Ref.	Ref.	Ref.		Ref.	Ref.	Ref.	
Black	0.827	(0.738, 0.925)	0.001		0.951	(0.862, 1.049)	0.313	
Asian	0.43	(0.32, 0.579)	<0.001		0.831	(0.701, 0.985)	0.033	
Hispanic	0.79	(0.705, 0.885)	<0.001		0.943	(0.859, 1.035)	0.214	
Unknown/missing	1.29	(1.007, 1.653)	0.043		1.053	(0.842, 1.316)	0.653	
Baseline Comorbidities								
Congestive Heart Failure	1.028	(0.936, 1.129)	0.559		1.11	(1.027, 1.2)	0.008	
Hypertension	1.113	(0.971, 1.275)	0.124		1.072	(0.959, 1.199)	0.222	
Hyperlipidemia/ Hypercholesteremia	1.063	(0.943, 1.197)	0.319		1.124	(1.013, 1.249)	0.028	
Chronic Kidney Disease	0.995	(0.877, 1.128)	0.934		0.854	(0.767, 0.952)	0.004	
Serious Hypoglyc. Event	1.056	(0.711, 1.566)	0.789		1.447	(1.07, 1.956)	0.016	
Serious Hyperglyc. Event	1.166	(0.813, 1.672)	0.404		1.172	(0.861, 1.595)	0.313	
Diabetic Nephropathy	1.215	(1.072, 1.376)	0.002		1.135	(1.02, 1.263)	0.02	
Diabetic Neuropathy	1.277	(1.175, 1.388)	<0.001		1.079	(1.004, 1.159)	0.039	
Diabetic Retinopathy	1.152	(1.022, 1.298)	0.021		1.09	(0.985, 1.207)	0.096	
Foot Ulcers	0.888	(0.804, 0.981)	0.019		0.923	(0.851, 1.002)	0.055	
End Stage Renal Disease	1.183	(0.848, 1.65)	0.322		1.024	(0.751, 1.397)	0.88	
Obesity	1.604	(1.484, 1.733)	<0.001		1.048	(0.979, 1.123)	0.175	
Smoker	0.906	(0.836, 0.983)	0.017		0.907	(0.847, 0.971)	0.005	
Unstable Angina	1.065	(0.904, 1.254)	0.453		0.946	(0.818, 1.094)	0.452	
Peripheral Artery Disease	0.928	(0.841, 1.024)	0.136		0.909	(0.838, 0.986)	0.021	
Myocardial Infarction	1.032	(0.941, 1.132)	0.499		1.065	(0.988, 1.148)	0.103	
CABG	1.066	(0.958, 1.185)	0.243		1.144	(1.052, 1.244)	0.002	
PCI	1.141	(1.039, 1.253)	0.006		1.295	(1.2, 1.397)	<0.001	
Stroke	0.879	(0.788, 0.981)	0.021		0.767	(0.698, 0.844)	<0.001	

OOP = Out-of-pocket

© 2023 Luo J et al. *JAMA Network Open.*

eTable 5. Full cox proportional hazards model output, Model #4

	GLP-1a initiation				SGLT2i initiation			
**Variable**	**Hazard ratio**	**95% Conf. Interval**	**P-value**	**Omnibus P-value**	**Hazard ratio**	**95% Conf. Interval**	**P-value**	**Omnibus P-value**
Quartile of OOP Costs				0.006				<0.001
1	Ref.	Ref.	Ref.		Ref.	Ref.	Ref.	
2	1.042	(0.944, 1.151)	0.414		0.947	(0.868, 1.032)	0.213	
3	0.929	(0.834, 1.034)	0.177		0.913	(0.834, 0.999)	0.047	
4	0.864	(0.773, 0.966)	0.01		0.804	(0.733, 0.881)	<0.001	
Age in years	0.943	(0.939, 0.947)	<0.001		0.958	(0.955, 0.962)	<0.001	
Sex				<0.001				<0.001
Male	Ref.	Ref.	Ref.		Ref.	Ref.	Ref.	
Female	1.202	(1.113, 1.299)	<0.001		0.777	(0.726, 0.831)	<0.001	
Unknown	0	(0, Inf)	0.983		0	(0, 3.2e+263)	0.979	
Geographical Division				<0.001				0.001
East North Central	Ref.	Ref.	Ref.		Ref.	Ref.	Ref.	
East South Central	1.266	(1.043, 1.536)	0.017		1.022	(0.856, 1.22)	0.808	
Middle Atlantic	0.939	(0.778, 1.132)	0.509		1.025	(0.883, 1.19)	0.746	
Mountain	0.83	(0.701, 0.982)	0.03		0.85	(0.739, 0.978)	0.023	
New England	0.932	(0.732, 1.187)	0.567		1.122	(0.932, 1.351)	0.223	
Pacific	0.817	(0.698, 0.956)	0.012		0.977	(0.863, 1.106)	0.714	
South Atlantic	0.995	(0.872, 1.135)	0.937		0.969	(0.868, 1.082)	0.577	
Unknown	0.686	(0.22, 2.14)	0.516		0.591	(0.22, 1.582)	0.295	
West North Central	1.17	(0.981, 1.395)	0.08		1.088	(0.933, 1.269)	0.28	
West South Central	0.946	(0.82, 1.093)	0.452		0.83	(0.734, 0.939)	0.003	
Metformin fill year				<0.001				<0.001
2017	Ref.	Ref.	Ref.		Ref.	Ref.	Ref.	
2018	1.205	(1.098, 1.323)	<0.001		1.209	(1.116, 1.31)	<0.001	
2019	1.582	(1.381, 1.812)	<0.001		2.128	(1.9, 2.385)	<0.001	
2020	2.09	(1.783, 2.449)	<0.001		3.42	(3.008, 3.888)	<0.001	
Education				0.541				0.15
<12th grade	Ref.	Ref.	Ref.		Ref.	Ref.	Ref.	
High school diploma	1.135	(0.718, 1.795)	0.589		0.844	(0.606, 1.175)	0.315	
<Bachelor’s degree	1.152	(0.727, 1.825)	0.546		0.86	(0.617, 1.2)	0.375	
≥Bachelor’s degree	1.122	(0.696, 1.808)	0.636		0.966	(0.683, 1.365)	0.844	
Unknown/missing	0.824	(0.45, 1.509)	0.531		1.009	(0.638, 1.597)	0.968	
Household income				0.001				0.036
<$40K	Ref.	Ref.	Ref.		Ref.	Ref.	Ref.	
$40K-$49K	1.131	(0.994, 1.288)	0.062		1.06	(0.948, 1.185)	0.305	
$50K-$59K	0.961	(0.834, 1.106)	0.576		1.111	(0.994, 1.241)	0.064	
$60K-$74K	1.093	(0.96, 1.246)	0.179		1.032	(0.925, 1.152)	0.573	
$75K-$99K	1.192	(1.055, 1.347)	0.005		1.128	(1.018, 1.25)	0.021	
$100K+	1.202	(1.057, 1.366)	0.005		1.065	(0.956, 1.187)	0.25	
Unknown/missing	0.797	(0.635, 1)	0.05		0.832	(0.689, 1.005)	0.057	
Race				<0.001				0.159

© 2023 Luo J et al. *JAMA Network Open.*

White	Ref.	Ref.	Ref.		Ref.	Ref.	Ref.	
Black	0.828	(0.74, 0.927)	0.001		0.954	(0.865, 1.052)	0.342	
Asian	0.43	(0.32, 0.579)	<0.001		0.83	(0.7, 0.984)	0.032	
Hispanic	0.789	(0.704, 0.884)	<0.001		0.943	(0.859, 1.034)	0.211	
Unknown/missing	1.296	(1.012, 1.66)	0.04		1.057	(0.845, 1.321)	0.628	
Baseline Comorbidities								
Congestive Heart Failure	1.039	(0.946, 1.141)	0.426		1.12	(1.036, 1.211)	0.004	
Hypertension	1.111	(0.97, 1.273)	0.13		1.073	(0.959, 1.199)	0.218	
Hyperlipidemia/ Hypercholesteremia	1.068	(0.948, 1.203)	0.282		1.13	(1.017, 1.254)	0.023	
Chronic Kidney Disease	1.001	(0.882, 1.135)	0.993		0.859	(0.77, 0.957)	0.006	
Serious Hypoglyc. Event	1.057	(0.712, 1.568)	0.784		1.454	(1.075, 1.965)	0.015	
Serious Hyperglyc. Event	1.171	(0.817, 1.68)	0.39		1.171	(0.861, 1.594)	0.314	
Diabetic Nephropathy	1.221	(1.077, 1.383)	0.002		1.137	(1.022, 1.265)	0.018	
Diabetic Neuropathy	1.304	(1.199, 1.417)	<0.001		1.097	(1.021, 1.179)	0.012	
Diabetic Retinopathy	1.161	(1.03, 1.309)	0.014		1.098	(0.992, 1.216)	0.072	
Foot Ulcers	0.893	(0.809, 0.986)	0.025		0.928	(0.855, 1.007)	0.072	
End Stage Renal Disease	1.187	(0.851, 1.655)	0.313		1.026	(0.752, 1.4)	0.87	
Obesity	1.605	(1.485, 1.734)	<0.001		1.05	(0.981, 1.125)	0.16	
Smoker	0.918	(0.847, 0.996)	0.039		0.918	(0.857, 0.983)	0.014	
Unstable Angina	1.056	(0.897, 1.244)	0.511		0.943	(0.815, 1.09)	0.425	
Peripheral Artery Disease	0.932	(0.845, 1.029)	0.162		0.913	(0.842, 0.99)	0.028	
Myocardial Infarction	1.03	(0.939, 1.13)	0.53		1.062	(0.985, 1.145)	0.118	
CABG	1.07	(0.962, 1.191)	0.211		1.146	(1.054, 1.247)	0.001	
PCI	1.14	(1.038, 1.252)	0.006		1.293	(1.198, 1.395)	<0.001	
Stroke	0.883	(0.792, 0.985)	0.026		0.77	(0.7, 0.847)	<0.001	
Insurance type								
Commercial	Ref.	Ref.	Ref.		Ref.	Ref.	Ref.	
Medicare	0.759	(0.68, 0.847)	<0.001		0.74	(0.671, 0.815)	<0.001	

OOP = Out-of-pocket; Ref.=Reference; Hyperglyc. = Hyperglycemic

© 2023 Luo J et al. *JAMA Network Open.*

eTable 6. Full cox proportional hazards model output, Model #5

	GLP-1a initiation				SGLT2i initiation			
**Variable**	**Hazard ratio**	**95% Conf. Interval**	**P-value**	**Omnibus P-value**	**Hazard ratio**	**95% Conf. Interval**	**P-value**	**Omnibus P-value**
Quartile of OOP Costs				0.006				<0.001
1	Ref.	Ref.	Ref.		Ref.	Ref.	Ref.	
2	1.041	(0.943, 1.15)	0.427		0.946	(0.868, 1.032)	0.21	
3	0.929	(0.835, 1.034)	0.18		0.913	(0.835, 0.999)	0.048	
4	0.864	(0.773, 0.966)	0.01		0.803	(0.733, 0.881)	<0.001	
Age in years	0.944	(0.94, 0.948)	<0.001		0.958	(0.955, 0.962)	<0.001	
Sex				<0.001				<0.001
Male	Ref.	Ref.	Ref.		Ref.	Ref.	Ref.	
Female	1.194	(1.106, 1.29)	<0.001		0.775	(0.724, 0.829)	<0.001	
Unknown	0	(0, Inf)	0.983		0	(0, 4.8e+263)	0.979	
Geographical Division				<0.001				0.001
East North Central	Ref.	Ref.	Ref.		Ref.	Ref.	Ref.	
East South Central	1.275	(1.051, 1.547)	0.014		1.025	(0.859, 1.224)	0.781	
Middle Atlantic	0.924	(0.766, 1.115)	0.412		1.012	(0.871, 1.175)	0.877	
Mountain	0.832	(0.703, 0.985)	0.033		0.852	(0.74, 0.98)	0.025	
New England	0.923	(0.724, 1.175)	0.515		1.119	(0.929, 1.347)	0.237	
Pacific	0.82	(0.701, 0.96)	0.013		0.977	(0.863, 1.106)	0.714	
South Atlantic	0.995	(0.872, 1.135)	0.936		0.967	(0.866, 1.08)	0.548	
Unknown	0.687	(0.22, 2.145)	0.519		0.591	(0.22, 1.583)	0.295	
West North Central	1.178	(0.988, 1.405)	0.067		1.09	(0.935, 1.271)	0.271	
West South Central	0.954	(0.827, 1.102)	0.523		0.831	(0.735, 0.94)	0.003	
Metformin fill year				<0.001				<0.001
2017	Ref.	Ref.	Ref.		Ref.	Ref.	Ref.	
2018	1.207	(1.099, 1.326)	<0.001		1.209	(1.116, 1.311)	<0.001	
2019	1.573	(1.373, 1.802)	<0.001		2.128	(1.899, 2.385)	<0.001	
2020	2.083	(1.777, 2.441)	<0.001		3.423	(3.01, 3.893)	<0.001	
Education				0.529				0.186
<12th grade	Ref.	Ref.	Ref.		Ref.	Ref.	Ref.	
High school diploma	1.146	(0.725, 1.813)	0.559		0.847	(0.609, 1.18)	0.326	

© 2023 Luo J et al. *JAMA Network Open.*

<Bachelor's degree	1.16	(0.732, 1.838)	0.527		0.863	(0.619, 1.203)	0.385	
≥Bachelor's degree	1.119	(0.695, 1.804)	0.644		0.963	(0.681, 1.361)	0.831	
Unknown/missing	0.833	(0.455, 1.526)	0.554		1.009	(0.638, 1.597)	0.968	
Household income				0.001				0.044
<$40K	Ref.	Ref.	Ref.		Ref.	Ref.	Ref.	
$40K-$49K	1.13	(0.992, 1.286)	0.065		1.058	(0.947, 1.183)	0.318	
$50K-$59K	0.957	(0.831, 1.103)	0.546		1.108	(0.992, 1.238)	0.07	
$60K-$74K	1.092	(0.959, 1.244)	0.185		1.031	(0.924, 1.151)	0.584	
$75K-$99K	1.187	(1.05, 1.34)	0.006		1.125	(1.015, 1.246)	0.025	
$100K+	1.192	(1.048, 1.355)	0.007		1.058	(0.95, 1.179)	0.306	
Unknown/missing	0.797	(0.635, 1)	0.05		0.834	(0.69, 1.006)	0.058	
Race				<0.001				0.142
White	Ref.	Ref.	Ref.		Ref.	Ref.	Ref.	
Black	0.829	(0.741, 0.928)	0.001		0.953	(0.865, 1.051)	0.337	
Asian	0.426	(0.317, 0.574)	<0.001		0.826	(0.697, 0.98)	0.028	
Hispanic	0.79	(0.705, 0.886)	<0.001		0.941	(0.858, 1.033)	0.201	
Unknown/missing	1.29	(1.008, 1.652)	0.043		1.055	(0.844, 1.319)	0.638	
Baseline Comorbidities								
Congestive Heart Failure	1.038	(0.945, 1.14)	0.434		1.119	(1.035, 1.21)	0.005	
Hypertension	1.112	(0.971, 1.275)	0.125		1.073	(0.96, 1.2)	0.214	
Hyperlipidemia/ Hypercholesteremia	1.062	(0.942, 1.197)	0.324		1.126	(1.013, 1.25)	0.027	
Chronic Kidney Disease	1.002	(0.884, 1.137)	0.969		0.859	(0.771, 0.957)	0.006	
Serious Hypoglyc. Event	1.023	(0.689, 1.518)	0.911		1.424	(1.053, 1.925)	0.022	
Serious Hyperglyc. Event	1.173	(0.818, 1.682)	0.386		1.174	(0.863, 1.598)	0.308	
Diabetic Nephropathy	1.213	(1.071, 1.375)	0.002		1.136	(1.021, 1.263)	0.019	
Diabetic Neuropathy	1.295	(1.191, 1.408)	<0.001		1.095	(1.018, 1.176)	0.014	
Diabetic Retinopathy	1.153	(1.023, 1.3)	0.02		1.094	(0.988, 1.211)	0.085	
Foot Ulcers	0.897	(0.812, 0.99)	0.031		0.929	(0.856, 1.008)	0.077	

© 2023 Luo J et al. *JAMA Network Open.*

End Stage Renal Disease	1.189	(0.853, 1.659)	0.307		1.023	(0.75, 1.395)	0.887	
Obesity	1.599	(1.48, 1.728)	<0.001		1.049	(0.98, 1.124)	0.166	
Smoker	0.921	(0.849, 0.999)	0.047		0.919	(0.859, 0.984)	0.016	
Unstable Angina	1.058	(0.898, 1.246)	0.499		0.942	(0.815, 1.089)	0.42	
Peripheral Artery Disease	0.933	(0.845, 1.029)	0.165		0.914	(0.843, 0.992)	0.03	
Myocardial Infarction	1.031	(0.939, 1.13)	0.524		1.062	(0.985, 1.145)	0.119	
CABG	1.067	(0.959, 1.187)	0.231		1.144	(1.052, 1.244)	0.002	
PCI	1.14	(1.038, 1.252)	0.006		1.29	(1.195, 1.391)	<0.001	
Stroke	0.882	(0.79, 0.984)	0.024		0.771	(0.701, 0.848)	<0.001	
Insurance type								
Commercial	Ref.	Ref.	Ref.		Ref.	Ref.	Ref.	
Medicare	0.765	(0.685, 0.854)	<0.001		0.744	(0.675, 0.82)	<0.001	
Provider type				<0.001				0.018
Fam. Prac./Internal Med.	Ref.	Ref.	Ref.		Ref.	Ref.	Ref.	
Endocrinology	1.63	(1.351, 1.967)	<0.001		1.343	(1.126, 1.601)	0.001	
Cardiology	0.983	(0.735, 1.316)	0.911		1.173	(0.951, 1.447)	0.135	
Unknown	0.974	(0.855, 1.108)	0.685		1	(0.898, 1.112)	0.994	
Other	1.079	(0.969, 1.202)	0.165		0.998	(0.908, 1.096)	0.964	

OOP = Out-of-pocket; Ref.=Reference; Hyperglyc. = Hyperglycemic; Hypoglyc. = Hypoglycemic; Fam. Prac./Internal Med.= Family Practice/Internal Medicine

© 2023 Luo J et al. *JAMA Network Open.*

eTable 7. Full cox proportional hazards model output, Model #6

	GLP-1a initiation				SGLT2i initiation			
**Variable**	**Hazard ratio**	**95% Conf. Interval**	**P-value**	**Omnibus P-value**	**Hazard ratio**	**95% Conf. Interval**	**P-value**	**Omnibus P-value**
Quartile of OOP Costs				0.007				<0.001
1	Ref.	Ref.	Ref.		Ref.	Ref.	Ref.	
2	1.05	(0.95, 1.16)	0.341		0.947	(0.868, 1.032)	0.215	
3	0.937	(0.842, 1.043)	0.234		0.914	(0.835, 1)	0.05	
4	0.871	(0.779, 0.974)	0.015		0.804	(0.733, 0.881)	<0.001	
Age in years	0.944	(0.94, 0.948)	<0.001		0.958	(0.955, 0.962)	<0.001	
Sex				<0.001				<0.001
Male	Ref.	Ref.	Ref.		Ref.	Ref.	Ref.	
Female	1.196	(1.107, 1.292)	<0.001		0.775	(0.724, 0.829)	<0.001	
Unknown	0	(0, Inf)	0.983		0	(0, 4.1e+263)	0.979	
Geographical Division				0.002				0.001
East North Central	Ref.	Ref.	Ref.		Ref.	Ref.	Ref.	
East South Central	1.27	(1.047, 1.541)	0.015		1.025	(0.859, 1.224)	0.783	
Middle Atlantic	0.933	(0.773, 1.126)	0.468		1.013	(0.873, 1.177)	0.861	
Mountain	0.852	(0.719, 1.009)	0.064		0.855	(0.743, 0.984)	0.029	
New England	0.914	(0.717, 1.164)	0.466		1.117	(0.928, 1.345)	0.242	
Pacific	0.838	(0.715, 0.981)	0.028		0.98	(0.865, 1.11)	0.754	
South Atlantic	1.009	(0.884, 1.152)	0.894		0.969	(0.867, 1.082)	0.576	
Unknown	0.71	(0.227, 2.216)	0.555		0.593	(0.221, 1.59)	0.299	
West North Central	1.152	(0.966, 1.375)	0.115		1.086	(0.931, 1.268)	0.293	
West South Central	0.964	(0.835, 1.113)	0.615		0.832	(0.736, 0.941)	0.003	
Metformin fill year				<0.001				<0.001
2017	Ref.	Ref.	Ref.		Ref.	Ref.	Ref.	
2018	1.208	(1.1, 1.327)	<0.001		1.21	(1.116, 1.311)	<0.001	
2019	1.566	(1.367, 1.794)	<0.001		2.127	(1.898, 2.383)	<0.001	
2020	2.076	(1.771, 2.433)	<0.001		3.421	(3.008, 3.89)	<0.001	
Education				0.544				0.185
<12th grade	Ref.	Ref.	Ref.		Ref.	Ref.	Ref.	
High school diploma	1.151	(0.728, 1.82)	0.548		0.847	(0.609, 1.18)	0.327	

© 2023 Luo J et al. *JAMA Network Open.*

<Bachelor’s degree	1.166	(0.736, 1.847)	0.513		0.863	(0.619, 1.204)	0.386	
≥Bachelor’s degree	1.125	(0.698, 1.813)	0.628		0.963	(0.681, 1.362)	0.832	
Unknown/missing	0.843	(0.461, 1.544)	0.581		1.011	(0.639, 1.599)	0.963	
Household income				0.001				0.042
<$40K	Ref.	Ref.	Ref.		Ref.	Ref.	Ref.	
$40K-$49K	1.129	(0.992, 1.286)	0.066		1.058	(0.947, 1.183)	0.317	
$50K-$59K	0.96	(0.834, 1.106)	0.574		1.108	(0.992, 1.238)	0.069	
$60K-$74K	1.096	(0.962, 1.248)	0.169		1.032	(0.924, 1.151)	0.579	
$75K-$99K	1.196	(1.059, 1.351)	0.004		1.126	(1.016, 1.248)	0.024	
$100K+	1.202	(1.057, 1.366)	0.005		1.059	(0.951, 1.18)	0.296	
Unknown/missing	0.793	(0.632, 0.995)	0.046		0.833	(0.69, 1.006)	0.057	
Race				<0.001				0.154
White	Ref.	Ref.	Ref.		Ref.	Ref.	Ref.	
Black	0.828	(0.74, 0.927)	0.001		0.953	(0.864, 1.051)	0.334	
Asian	0.431	(0.321, 0.58)	<0.001		0.828	(0.698, 0.982)	0.03	
Hispanic	0.805	(0.718, 0.903)	<0.001		0.944	(0.86, 1.036)	0.224	
Unknown/missing	1.299	(1.015, 1.664)	0.038		1.056	(0.845, 1.32)	0.632	1.299
Baseline Comorbidities								
Congestive Heart Failure	1.034	(0.941, 1.135)	0.488		1.119	(1.035, 1.209)	0.005	
Hypertension	1.109	(0.968, 1.271)	0.135		1.073	(0.96, 1.2)	0.215	
Hyperlipidemia/ Hypercholesteremia	1.072	(0.951, 1.208)	0.254		1.127	(1.015, 1.252)	0.025	
Chronic Kidney Disease	1.008	(0.889, 1.144)	0.897		0.86	(0.771, 0.958)	0.006	
Serious Hypoglyc. Event	1.025	(0.691, 1.522)	0.901		1.424	(1.054, 1.926)	0.021	
Serious Hyperglyc. Event	1.173	(0.818, 1.682)	0.386		1.174	(0.862, 1.597)	0.309	
Diabetic Nephropathy	1.218	(1.075, 1.38)	0.002		1.136	(1.021, 1.264)	0.019	
Diabetic Neuropathy	1.299	(1.194, 1.412)	<0.001		1.095	(1.019, 1.177)	0.014	
Diabetic Retinopathy	1.157	(1.027, 1.305)	0.017		1.094	(0.988, 1.212)	0.083	
Foot Ulcers	0.902	(0.816, 0.996)	0.041		0.93	(0.856, 1.009)	0.08	
End Stage Renal Disease	1.188	(0.852, 1.657)	0.31		1.022	(0.75, 1.395)	0.889	

© 2023 Luo J et al. *JAMA Network Open.*

Obesity	1.603	(1.483, 1.732)	<0.001		1.05	(0.981, 1.124)	0.162	
Smoker	0.918	(0.846, 0.995)	0.038		0.919	(0.858, 0.984)	0.015	
Unstable Angina	1.057	(0.898, 1.245)	0.504		0.942	(0.815, 1.089)	0.419	
Peripheral Artery Disease	0.937	(0.849, 1.034)	0.193		0.915	(0.843, 0.992)	0.032	
Myocardial Infarction	1.03	(0.939, 1.13)	0.535		1.062	(0.985, 1.144)	0.12	
CABG	1.064	(0.957, 1.184)	0.252		1.143	(1.051, 1.244)	0.002	
PCI	1.135	(1.033, 1.247)	0.008		1.289	(1.194, 1.391)	<0.001	
Stroke	0.879	(0.788, 0.981)	0.021		0.771	(0.701, 0.848)	<0.001	
Insurance type								
Commercial	Ref.	Ref.	Ref.		Ref.	Ref.	Ref.	
Medicare	0.772	(0.692, 0.863)	<0.001		0.745	(0.676, 0.821)	<0.001	
Provider type				<0.001				0.018
Fam. Prac./Internal Med.	Ref.	Ref.	Ref.		Ref.	Ref.	Ref.	
Endocrinology	1.636	(1.356, 1.974)	<0.001		1.344	(1.127, 1.602)	0.001	
Cardiology	0.981	(0.733, 1.313)	0.896		1.173	(0.951, 1.446)	0.136	
Unknown	0.971	(0.853, 1.106)	0.659		0.999	(0.898, 1.112)	0.989	
Other	1.074	(0.964, 1.197)	0.193		0.997	(0.908, 1.095)	0.952	
HbA1c at Baseline	0.893	(0.826, 0.966)	0.005		0.983	(0.921, 1.049)	0.606	

OOP = Out-of-pocket; Ref.=Reference; Hyperglyc. = Hyperglycemic; Hypoglyc. = Hypoglycemic; Fam. Prac./Internal Med.= Family Practice/Internal Medicine

© 2023 Luo J et al. *JAMA Network Open.*

eFigure 1. Cumulative incidence curve for statin initiation stratified by Q1 through Q4 of OOP costs for GLP-1a

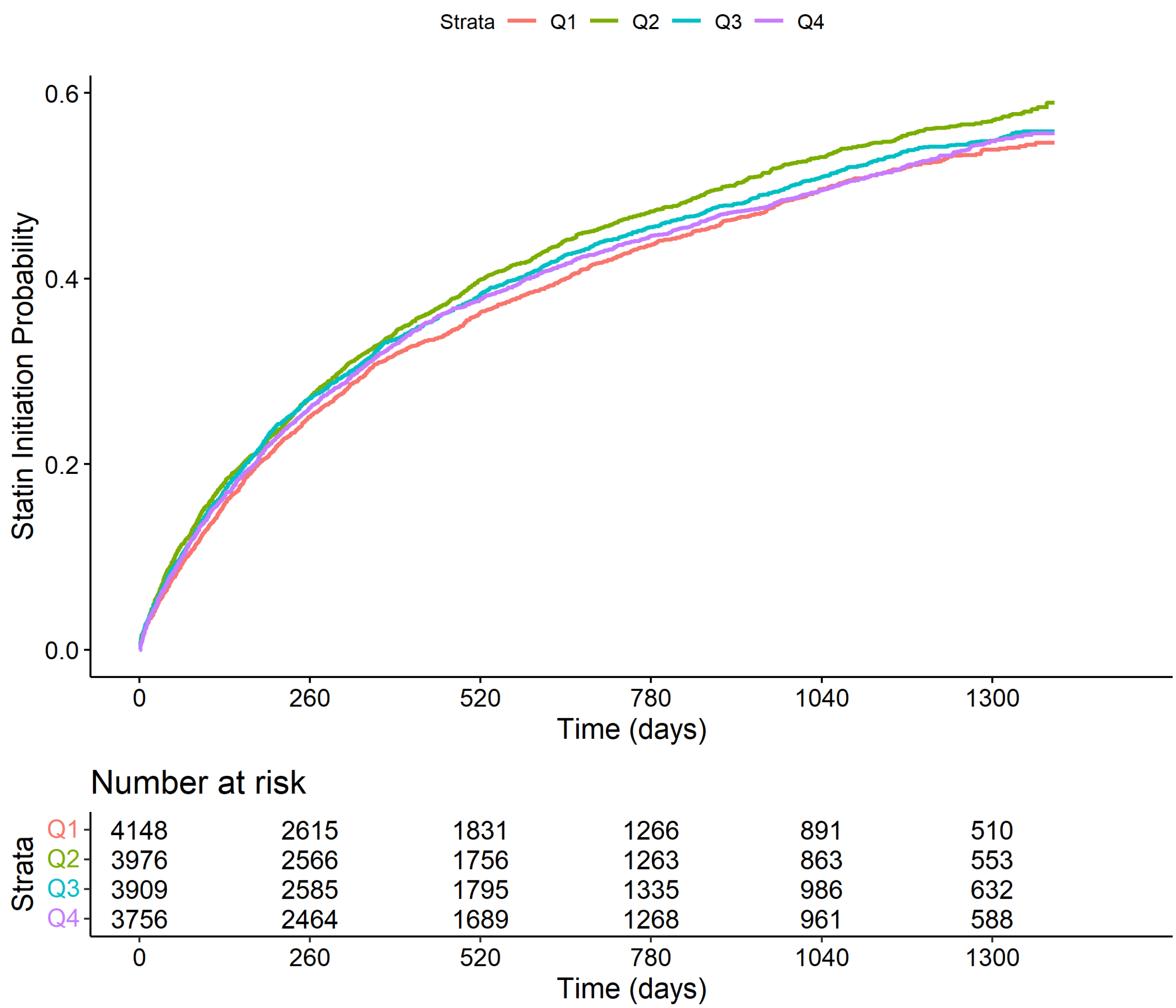

© 2023 Luo J et al. *JAMA Network Open.*

eFigure 2. Cumulative incidence curve for statin initiation stratified by Q1 through Q4 of OOP costs for SGLT2i.

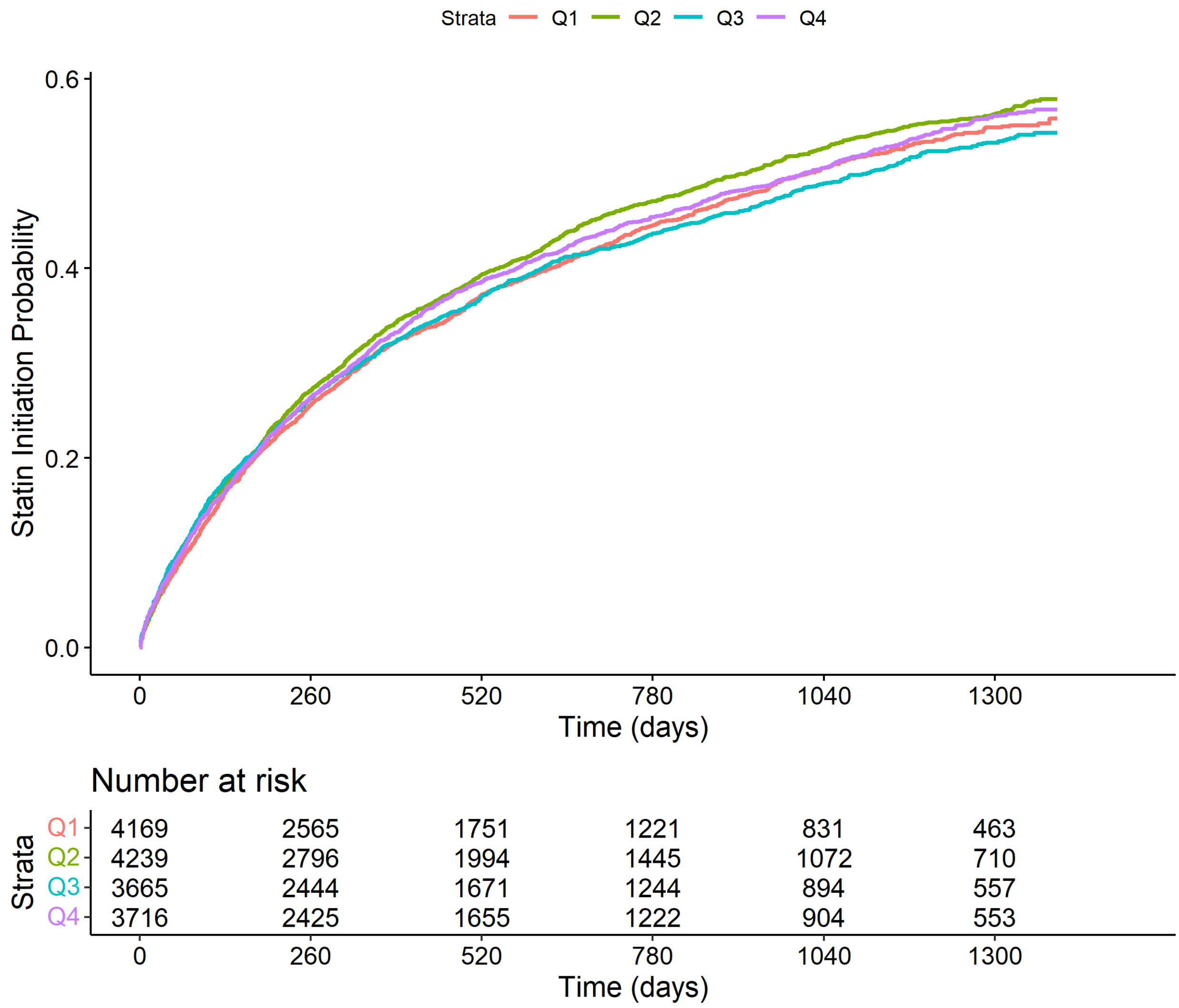

© 2023 Luo J et al. *JAMA Network Open.*

eTable 8: Hazard ratio of initiating a statin comparing the highest versus the lowest quartile of OOP costs for GLP-1 receptor agonists or SGLT2 inhibitors.

Model #	Description of model adjustments	Statin initiation Hazard Ratio (95% Confidence Interval), Q4 vs Q1 OOP costs for GLP-1 agonists	Statin initiation Hazard Ratio (95% Confidence Interval), Q4 vs Q1 OOP Costs for SGLT2 inhibitors
1	Quartile of OOP costs only	1.04 (0.97 to 1.11)	1.04 (0.97 to 1.11)
2	Model 1+demographics*	1.08 (1.01 to 1.16)	1.11 (1.04 to 1.19)
3	Model 2+comorbidities**	1.09 (1.01 to 1.17)	1.12 (1.04 to 1.20)
4	Model 3+insurance type	1.08 (1.01 to 1.16)	1.10 (1.03 to 1.18)
5	Model 4+provider type	1.09 (1.01 to 1.17)	1.10 (1.03 to 1.19)
6	Model 5+has HbA1c lab result	1.08 (1.01 to 1.16)	1.10 (1.03 to 1.18)

OOP = Out-of-pocket

*age, gender, division, metformin fill year, education level, household income range, race

**heart failure, hypertension, hyperlipidemia, chronic kidney disease, serious hypoglycemic events, serious hyperglycemic events, diabetic nephropathy, diabetic neuropathy, diabetic retinopathy, foot ulcers, end state renal disease, obesity, smoking status, unstable angina, peripheral artery disease, myocardial infarction, coronary artery bypass graft, Percutaneous coronary intervention, stroke

© 2023 Luo J et al. *JAMA Network Open.*
